# From the Compact City to the X-Minute neighborhood: A Systematic Review of the Health and Wellbeing Impacts of Sustainable Urban Development Models (SUDMs) on Women

**DOI:** 10.1007/s11524-025-00990-z

**Published:** 2025-09-11

**Authors:** Amy Stevenson, Vicki Ponce Hardy, Nick Bailey, Jaime Toney, Jonathan R. Olsen, Petra Meier

**Affiliations:** 1 Urban Big Data Centre, University of Glasgow, Glasgow, Scotland, UK; 2 MRC/CSO Social and Public Health Sciences Unit, University of Glasgow, Glasgow, Scotland, UK; 3 School of Geographical and Earth Sciences, University of Glasgow, Glasgow, Scotland, UK

**Keywords:** Feminist urbanism, Sustainability, 20-minute neighborhoods, Women's health, Gendered cities

## Abstract

Throughout the past 50 years, sustainable urban development models (SUDMs) have been introduced in cities across the world with the intention of limiting environmental air pollution and, more recently, greenhouse gas emissions. However, the health and wellbeing impacts that these interventions have had on different demographic groups are not well understood. Feminist urbanists have often critiqued hierarchical and non-participatory approaches to urban design for the detrimental impact they may have on women and minority groups. With x-minute neighborhood policies gaining popularity in urban planning across the world, gathering evidence on the potential gendered health and wellbeing inequalities impacts of these policies is a salient issue. Our research questions were as follows: (1) In the existing literature, what is known about the health and wellbeing impacts of SUDMs on women? (2) What mechanistic pathways are outlined in existing literature from SUDMs to gendered health outcomes? This review searched Medline, SCOPUS, Science Citation Index Expanded, Social Sciences Citation Index, and ASSIA. A broad range of outcomes was included in the search, from physical and mental health and wellbeing to health behaviors. We searched for empirical papers published in English before January 1st, 2024, without limiting the search by year or country of publication. Screening was performed on Rayyan with 15% of records double-screened. Critical appraisal was conducted using the AXIS tool for cross-sectional studies and CASP cohort checklist for longitudinal studies. Narrative synthesis was used to explore results in depth, with an effect-direction plot used to visually summarize findings. The initial search returned 1263 records. After duplicates were removed, 1194 records remained for screening. Of these, 301 were included for full-text screening, with 25 included for data extraction. Most of the included papers explored associations between SUDMs and women’s physical activity. These relationships were typically positive, although some found no significant associations. Papers which explored the gendered mechanisms leading to outcomes tended to posit that having more convenient non-motorized access to a range of destinations on foot helped women to balance their paid and unpaid labor, leading to increased physical activity. Increased safety and reduced social isolation within SUDMs were also hypothesized as key contributing factors to women’s increased physical activity. We found that there are research gaps in relation to mental health and long-term physical health outcomes.

## Introduction

The majority of the world’s population currently resides in cities [1]. Current planning practices of most major cities are car-centric [2]. This increases the convenience of using cars in urban spaces as opposed to environmentally sustainable and active methods of travel such as walking, wheeling, cycling, or taking public transport. Sustainable urban development models (SUDMs) are urban planning frameworks designed to integrate the economic, social, physical and environmental systems in a city [3]. They present a significant opportunity to promote climate change mitigation and adaptation within an urban environment, whilst realising co-benefits for human health and wellbeing.

These co-benefits primarily result from urban planning priorities shifting to facilitate more active, rather than private vehicular, travel. Presently, car dependency leads to higher rates of particulate air pollution, accelerating climate change and contributing to an estimated 4.2 million premature deaths each year worldwide [4]. Car-centric cities also tend to be associated with lower rates of active travel [5]. Physical inactivity and loneliness, both associated with non-communicable diseases, are on the rise, especially in urban areas [6], highlighting a need to increase opportunities and incentives for regular physical activity and social interaction [7–9]. SUDMs align with this increased focus on ensuring that places where most people live provide opportunities for increased daily physical activity and social interaction [5, 10].

Urban planning models therefore have the potential to restructure the places in which we live to align more closely with collective aspirations to develop more sustainable, healthy societies around the world. However, cities are also sites of extreme inequality, and urban planning has often failed to embed this awareness into its models [11]. Indeed, systemic income, gender and racial inequalities have been reinforced by various urban planning models. For instance, Markley [12] has demonstrated that improving the walkability of neighborhoods can trigger gentrification, whilst Howard’s Garden City model is often cited as an example of urban planning which facilitated the perpetuation of traditional gender norms and the ‘male breadwinner’ model by relocating women out of the city [13, 14]. Several reviews have explored the relationship between urban models and health inequalities, with some focusing on particular populations and outcomes such as older people’s walking behavior [15]. Others have mapped either realized or hypothesized mechanistic pathways from interventions to outcomes [16]. Gendered health impacts have previously been explored in relation to particular features of urban spaces, such as greenspace [17, 18]. However, to our knowledge, this is the first review to critically analyse literature exploring the relationship between SUDMs as a whole and gendered health outcomes.

Some planners and practitioners are optimistic about the potential for proximity-based SUDMs to have positive impacts in relation to gender inequality [19–22]. Such models include x-minute neighborhoods (X-MNs), which aim to ensure convenient access to amenities that people use on a daily basis within a short walk from their home [23]. Indeed, a recent commitment to feminist city planning by Glasgow City Council in Scotland has claimed that walkability and proximity to services are key components of a gender-equal city [24]. This aligns with feminist urbanist literature, which argues that women’s needs should be substantively represented in urban planning, thereby reflecting and easing the execution of the disproportionate amount of unpaid care work undertaken by women [25, 26]. Table 1 outlines key tenets of feminist urbanism—descriptive and substantive representation, horizontality, and flexibility—and describes how each of these features relates to the planning process. Feminist urbanism also highlights the determining role of safety in women’s ability to traverse the city [27, 28]. Gargiulo et al. additionally suggest that women’s perceived unsafety in many urban contexts may lead to health inequalities between genders in the long term [28].

**Table 1:** Central tenets of feminist urbanism

Feature		Description
*Descriptive representation*	**General**	Planning as a field of work and study should represent wider demographics in society, including women and marginalized communities
	Participatory planning	Women and marginalized communities should be present in decision-making spaces [29]
	Intersectionality	The lived experience of women and marginalized groups should be acknowledged and incorporated into planning processes. Collecting data disaggregated by sex, ethnicity, class, disability and gender identity can enable this [20, 29]
*Substantive representation*	**General**	Cities should reflect the needs of people from a diverse range of backgrounds/demographics/identities who live in them
	Safety and accessibility	Cities should be designed to minimize gender-based violence and mobility challenges [30, 31]
	Care infrastructure	Women typically conduct a disproportionate share of caregiving duties. Access to childcare services, schools, community center , and care homes should be considered in planning [29]
	Economic equity	Cities should enable equitable access to workplaces and affordable housing [25]
*Horizontality*	**General**	Inclusive and community-led planning practices should be normalized. This should be reflected in the built environment
	Inclusive public spaces	Public spaces should represent diverse interests and needs in order to prevent de facto exclusion [30, 31]
	Community-led planning	A collective approach to planning within communities should be enabled across cities [29]
	Sustainability	Urban infrastructure should be resilient and benefit everyone equitably. Environmental justice should be considered within planning decisions [20]
*Flexibility*	**General**	Planning across cities should be flexible so as to accommodate the unique character and needs of different neighborhoods and people
	Mixed-use spaces	Dynamic land use that allows for live-work arrangements, flexible housing models, and evolving community needs [29]
	Responsive urbanism	City design should be adjusted based on lived experiences, community feedback, and shifting social patterns [20, 29]
	Inclusive mobility	Transport and urban infrastructure should accommodate flexible, multi-purpose journeys, such as trip-chaining [30, 31]

However, whether these claims are supported by empirical research, and how this specifically relates to SUDMs, has not yet been explored. A feminist urbanist perspective can help to frame questions relating to whether the benefits of future SUDMs such as X-MNs will be equitably shared across demographics. Marquet and Maciejewska [22], although acknowledging possible benefits of X-MNs, also outline three key cautionary points. First, the assumption that X-MNs will directly benefit women risks reinforcing regressive essentialist viewpoints which equate women with social reproductive labor. Second, there is a risk that women are perceived as a homogeneous group, failing to account for the diversity of women and their needs. This aligns with public health researchers who have similarly argued that it is important to adopt an intersectional systems thinking approach to assessing climate change mitigation and adaptation strategies [32]. Third, X-MNs do not automatically address well-established differences in men’s and women’s experiences of urban spaces which should be embedded into design priorities.

Although the X-MNs terminology is currently experiencing strong policy support, its concepts relating to human-centred, compact and sustainable urban planning have been central to related planning concepts that preceded the X-MN [33]. We can therefore draw on evidence from SUDMs preceding X-MNs, paying attention to gendered pathways to health outcomes in order to understand more about the potential resulting gendered health and wellbeing impacts.

This review aims to provide a gendered perspective on the potential health and wellbeing impacts of X-MNs, amidst their current policy support. Our research questions were as follows: (1) What is known in the literature about the gendered health and wellbeing impacts of SUDMs? (2) What mechanistic pathways are outlined that connect SUDMs to gendered health outcomes?

## Methods

The review followed the methodology outlined in the Cochrane Handbook for Systematic Reviews of Interventions [34]. This outlines standard procedure for defining research questions and inclusion criteria and a rigorous approach to data synthesis. We also used the Preferred Reporting Items for Systematic reviews and Meta-Analyses (PRISMA) reporting guidelines in accordance with the 2020 Checklist, which is included in Appendix 1 [35].

### Search and Screening

Information on the search strategy is provided in Appendix 2. The inclusion criteria were defined according to the PICOS—Population, Intervention, Comparator, Outcome, Study Design—structure and are listed in Table 2. The search was limited to peer-reviewed papers published in English prior to January 1 st, 2024.

**Table 2:** PICOS inclusion criteria

Category	Definition
Population	Global urban populations
Intervention	Any sustainable urban development intervention or model
Comparator	Any consideration of gender as a mechanism leading to health outcomes with sex or gender-disaggregated results, or studies focusing exclusively on women’s health outcomes
Outcome	Any physical health outcome (e.g. cardiovascular disease); any mental health outcome (e.g. depression); or health behaviors (e.g. walking)
Study Design	Any empirical peer-reviewed papers including cross-sectional and longitudinal studies

For interventions, we searched for known SUDM terms or labels [33, 36]. Definitions of these terms and their relation to sustainability are outlined in Appendix 3. Papers marked as looking at ‘x-minute neighborhood’ interventions included those which did not use this terminology but nonetheless described regions whereby residents could access amenities within a short distance from their home. Whilst there is significant overlap between the definition of ‘x-minute neighborhoods’ and ‘New urbanism’, papers with a focus on proximity would be categorized as ‘x-minute neighborhoods’ where they specified a distance from residences to destinations. This distance was typically defined in terms of the average walking time from home to destination, but in other cases was described in terms of literal distance, e.g. 800 m.

Since the topic is understudied and we wanted to understand the scope of the existing evidence base, we searched for and included a breadth of health outcomes related to physical and mental health and health behaviors. These are detailed in Appendix 2.

The relationship between place, health and gender is complex. This is further obfuscated by the use of inconsistent terminology in relation to sex and gender within health research. In simple terms, we might think of sex as a category important for understanding biological differences between males and females, whereas gender is a category used to understand the social and psychological determinants of health in self-identified men, women and people of other genders [37, 38]. In older health research, these terms are often used interchangeably. Since we were interested in women’s health outcomes specifically, we opted to include papers which disaggregated results by sex or gender and considered gendered pathways to health outcomes as well as those which had a women or female-only sample. This approach aligns with the World Health Organization’s guidance on gender-focused research [39].

We searched Medline, SCOPUS, Science Citation Index Expanded, Social Sciences Citation Index and ASSIA. We assessed the strategy’s effectiveness in identifying pre-defined target papers, amending the search until these papers showed up in at least one of the databases.

Results from the searches were saved to EndNote and then uploaded to Rayyan for screening. One author (AS) screened all the abstracts, whilst another (VPH) screened 15%. There was 95% agreement between reviewers at this stage, and where assessments differed (one marked ‘include’ whereas the other marked ‘maybe’), differences were discussed to reach consensus. At the full-text screening stage, there was 99% agreement between reviewers, and differences were resolved through discussion.

### Data Extraction

Data extraction was completed by the first author using Excel. Standard items extracted included the following: first author, year of publication, reference, link to paper, country of origin, study design, methods (quantitative, qualitative, modeling, specific type of review) and study objective(s). In addition, the following were also extracted: City/state of focus; Intervention—urban model; Outcomes and measures (all measures were included); Inequalities; Feminist urbanism components; Sample size; Gender, age, ethnicity, socioeconomic and disability distributions; Summary of results; Summary of inequalities impacts (if applicable); Limitations; and Mechanistic pathways.

### Critical Appraisal

Papers were assessed using the AXIS tool for cross-sectional studies [40], the Critical Appraisal Skills Programme (CASP) cohort checklist for longitudinal studies [41] and the CASP qualitative checklist to assess qualitative aspects of any cross-sectional studies [42]. Papers scoring 0–60% were assessed as ‘high risk of bias’; papers scoring 60–85% were assessed as having ‘some concerns’; and papers scoring 85–100% were assessed as ‘low risk of bias’.

### Data Synthesis

Due to the heterogeneity of measures used to assess interventions and health outcomes, and of sample sizes, narrative synthesis was considered more appropriate than meta-analysis for data synthesis. This also allowed for a more in-depth exploration of the mechanistic pathways within and between papers which would help to address the second research question. Guidance from the center for Reviews and Dissemination structured the narrative synthesis [43]. This consisted broadly of four stages: ‘developing a theory of how the intervention works, why and for whom; developing a preliminary synthesis of findings of included studies; exploring relationships within and between studies; and assessing the robustness of synthesis’ [43].

We also used effect direction plots—“a method of data visualisation in synthesis without meta-analysis” which provides information on effect direction for individual studies, as well as providing an overview of study characteristics and quality [44].

## Results

**Fig. 1:**
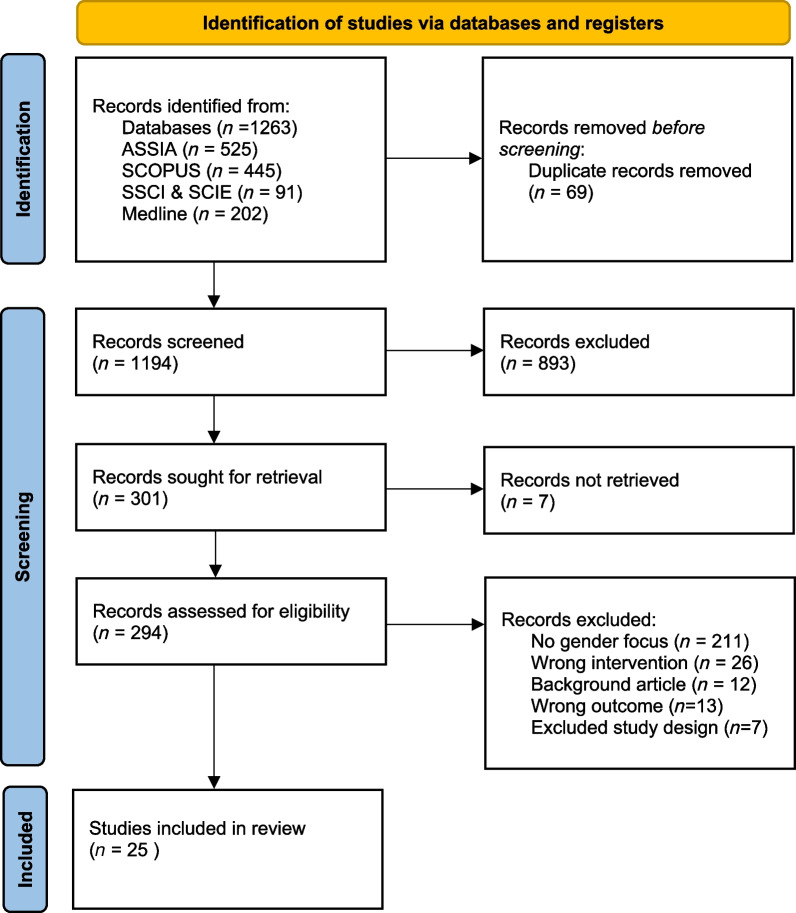
PRISMA flow diagram [35]

In the initial search, 1263 records were returned. After duplicates were removed, 1194 records remained for title/abstract screening with 301 subsequently included for full-text screening. The latter led to 25 papers being included in the review (Fig. 1).

**Fig. 2:**
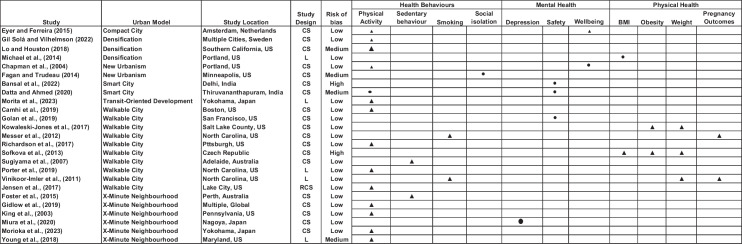
Effect-direction plot. Legend: study design—CS, cross-sectional; L, longitudinal; RCS, repeated cross-sectional. Effect direction: upward arrow ▲ = positive health impact, downward arrow ▼ = negative health impact, sideways arrow ◄► = mixed effects/conflicting findings, = no change. Sample size: final sample size (individuals) in intervention group large arrow▲ > 1000; medium arrow ▲ 100–999; small arrow ▲ < 100. Study quality: denoted by row pattern: high risk of bias:  ; some concerns:  ; low risk of bias:

Papers included in the review came from 7 countries: US (60%) [45–59], Japan (12%) [60–62], Australia and India (8%) [63–66], Czech Republic [67], Netherlands [68] and Sweden (4%) [69].They consisted of 19 (76%) cross-sectional studies, five (20%) longitudinal studies and one (4%) repeated cross-sectional study, as shown in Fig. 2. Of the 25 papers included, three (12%) were published in the decade between 2000 and 2009, 16 (64%) between 2010 and 2019 and six (24%) in the four years between 2020 and 2023, also detailed in Fig. 2.

### Use of Gender and Sex Terminology

There were differences in the reporting of data by gender and sex: 52% reported disaggregated results by ‘sex’, 48% by ‘gender’. Regardless of whether results were reported by gender or sex, gendered pathways were described in the discussion. As we synthesize published literature here, and only gendered pathways were described in the literature, we only refer to gender within this manuscript.

### Urban Concepts

Walkable cities and neighborhoods were explored in 40% of papers by using a version of a ‘walkability index’ [45, 48, 52–57, 64, 67]. Four (16%) of these utilized previously validated measures of walkability, namely the Walk Score (8%) [45, 48], neighborhood Environmental Walkability Scale (4%)[67] and PIN3 Environmental Audit Tool (4%) [55]; others (24%) created a new index deriving variables from either primary or secondary data sources. Three of the papers using a ‘walkability index’ used this to create a binary between ‘high’ and ‘low’ walkable areas (12%) [49, 51, 67]. Six papers (24%) explored X-MNs: five (20%) of these were measured by proximity to amenities and amenity diversity in relation to residence [59] Gidlow, King, Miura, Morioka, Young; one (4%) looked at a neighborhood classified as an X-MN relative to other conventional planning forms. Three papers (12%) explored densification using composite measures comprised of population density, amenity density and land-use mix [52, 54]. Two papers (8%) looked at new urbanism, one using an original New Urbanism Index [46] and the other by assessing a neighborhood in relation to the principles of new urbanism [47]. Two papers (8%) looked at smart cities: one looking at a city-wide approach to smart city planning [65], and the other focusing on specific interventions [66]. The compact city was explored in one paper (4%) by looking at a city-wide approach to compact city planning [68], whilst the transit-oriented development was studied in one paper (4%) by looking at a geographically limited intervention [61].

### Health Behaviours and Outcomes

Papers associated with each health-related outcome can be seen in Fig. 2. Across the 25 papers, 35 outcomes are reported, with some papers reporting on multiple outcomes. Of the included studies, over half (56%) reported physical activity as an outcome. Outcomes were measured either by self-reported walking behavior (24%), step count (12%), time spent engaged in moderate to vigorous physical activity (8%), observed walking behavior (4%) or self-reported sedentary time (8%). Smoking and social isolation were measured according to self-reported behavior and experience. Six papers (24%) reported on mental health outcomes. Depression was measured by the Edinburgh Post Partum Depression Scale in one paper (4%). Wellbeing was measured by the Quality of Life Index (4%) and self-defined emotional experience of daily travel journeys (4%). Safety was measured by self-reported feelings of safety (12%) and, as such, was categorized as a mental health outcome. Five papers reported on physical health outcomes. Of these, four (16%) papers calculated BMI from objective measures of height and weight, reporting on the following health outcomes: weight (12%), obesity (8%) and BMI (8%). Pregnancy outcomes were derived from large datasets and included: weight gain, low birth weight, pre-eclampsia, pre-term birth (8%). The effect direction of the health outcomes relative to the SUDMs is displayed in Fig. 2.

### Pathways

#### From SUDMs to Health Behaviours

In the reviewed studies, physical activity was the most commonly reported health behavior identified (56%). As cities incorporate more sustainable practices into their urban development, whether through improving walkability, increasing population density, or increasing proximity to local amenities within residential areas, there is a significantly positive association with women’s reported physical activity.

Reviewed studies outlined various pathways to physical activity. First, proximity to care infrastructure is associated with increased walking behavior in women. It is hypothesized that this is because increased proximity to care infrastructure allows women to more easily conduct social reproductive labor without the use of a car [50, 52, 53, 55, 59, 60, 62–64, 69]. These destinations were identified at various points as: department, discount and hardware stores; community infrastructure; and in some contexts, grocery stores [45, 46, 50, 55, 60, 63]. One paper found that new urbanism could help to reduce the burden of social reproductive labor on women by providing opportunities to outsource some tasks or conduct them closer to home and foster increased social interaction [47]. However, they caution that their findings suggest this applies only to a limited, already privileged group of women who were predisposed to live in areas which enable these behaviors.

Second, safety (conceptualized as a hypothesized or realized moderator rather than an outcome) was found to influence walking behavior. Walkability was assessed in several papers by a composite measure of intersection density, pavement width, and road traffic, known as the Walk Score (8%) [45, 48]. However, limitations to this were highlighted in one paper which found that these standard assessments of walkability excluded important safety markers which contributed to decisions on whether or not to walk in a given area, as described by an all-female sample [48]. This study found that crime rates, presence of encampments and street cleanliness were important markers of safety. As such, regions highlighted as having the highest walk scores were less likely to score highly on an alternative index which more closely reflects women’s conception of walkability. Other papers similarly hypothesized that increased walking behavior within proximity-based urban models could be partially attributed to improved feelings of safety [49, 56, 57].

One paper exploring the smart city model in India found that women’s walking behavior was not altered by technological innovations such as a digital safety app and a dedicated ‘she-corridor’ for women [66]. The latter comprised CCTV cameras, crime surveillance, accessible public toilets, breastfeeding centers and safe and accessible footpaths. However, women’s unequal access to digital infrastructure had not been accounted for and limited their ability to utilize the app, whilst the ‘she-corridor’ failed to account for the ubiquitous nature of violence against women in the city. This may explain why interventions did not alter women’s walking behavior.

Reduced smoking behavior during pregnancy was found to be associated with SUDMs in two studies [53, 57]. Authors hypothesize that the reduction in smoking behavior is due to improved walkability creating safer public spaces within which the exchange of knowledge of healthy behaviors such as reduced smoking during pregnancy may occur.

#### From SUDMs to Mental Health Outcomes

Outcomes documented in relation to mental health were as follows: postpartum depression (PPD), feelings of safety, and general wellbeing (24%). Here, evidence was mixed, with outcomes less likely to be positive than previously seen in relation to physical activity. The paper exploring PPD hypothesized that the presence of playgrounds within walking distance of the home enabled new mothers to spend more time in playgrounds with their children [62]. In turn, this afforded them more opportunities for social interaction with other new mothers. This subsequently created a greater sense of social support in the early months of motherhood, lowering the likelihood of developing PPD.

Papers which explored safety as an outcome of SUDMs measured self-reported feelings of safety. They did not find any significant relationship between SUDMs and safety, and argued that this is because interventions were not cognisant of wider systems of oppression [48, 65, 66]. Two papers found that, isolated from social and physical threats to women’s safety, digital interventions associated with the smart city model could not in themselves promote greater feelings of safety [65, 66]. They explain that this is due to unequal access to digital infrastructure and women requiring access to public spaces outside of the relatively safe ‘she-corridor’ [65, 66]. Meanwhile, another study found that standard walkability indices did not align with women’s concepts of safety [48]. As a general rule, areas which were highly walkable using standard assessments were places where women would feel too unsafe to utilize.

One paper looked at the relationship between new urbanism and women’s wellbeing, assessed using the New Urbanism Index and Quality of Life Index, respectively [46]. They found no significant associations between the two. Since women living in areas with high New Urbanism Index scores typically had lower incomes than those in areas with low New Urbanism Index scores, the authors hypothesize that the negative effects of low income overshadowed any positive effects of new urbanism.

#### From SUDMs to Physical Health Outcomes

Five papers (20%) considered the relationship between SUDMs and physical health outcomes. Michael et al.’s longitudinal study explored the relationship between interventions related to densification to facilitate more physical activity and its effect on the BMI of older white women [54]. No significant changes were observed, which the authors hypothesize was because the physical activity increase associated with the relevant changes in the built environment was of neither sufficient intensity nor duration to cause weight change which would be observed within the BMI metric. However, the author did acknowledge some elements of research design may have biased the findings towards the null hypothesis. Kowaleski-Jones et al. alternatively found that high walkability was associated with significantly lower BMI scores and rates of obesity in younger mothers, although the strength of the relationship was moderated by familial risk of obesity [51]. Sofkova et al.’s study may shed some light on this discrepancy beyond potential bias to the null in Michael et al.’s study [67]. These authors found that women living in highly walkable areas exhibited fewer health risks in terms of body composition than those in low walkable areas. However, when they explored results for different age groups, they found that women under 40 had significantly lower body fat when living in areas of high walkability, whereas there was no significance in this relationship for women aged 40 and above.

As previously mentioned, two studies explore the relationship between neighborhood walkability and smoking behavior during pregnancy [53, 57]. Both papers found that neighborhood walkability is associated with reduced smoking during pregnancy and subsequently better pregnancy outcomes. However, this was only true for white women and not for black and minority ethnic women included in the study. Unfortunately, neither study controlled for any measure of economic deprivation, and one of them hypothesized that this could be the reason for the ethnic disparities in results [53, 57].

### Intersectionality

**Fig. 3:**
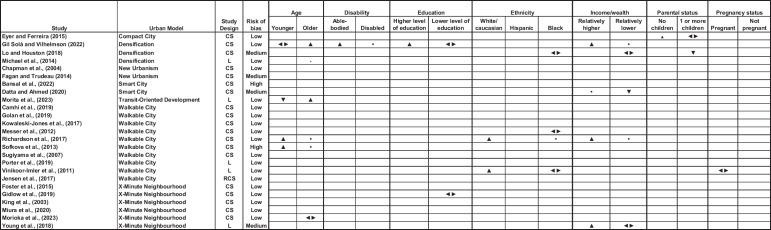
Effect-direction plot showing intersectional analyses in included studies. Legend: Study Design—CS, cross-sectional; L, longitudinal; RCS, repeated cross-sectional. Effect direction: upward arrow ▲ = positive health impact, downward arrow ▼ = negative health impact, sideways arrow ◄► = mixed effects/conflicting findings,  = no change. Study quality: denoted by row pattern: High risk of bias:  ; Some concerns:  ; Low risk of bias:

We found that 52% of papers considered gender in combination with other demographic characteristics. Twenty-four percent considered the intersection between age and gender [56, 61, 67, 69]; 20% considered the intersection between income and gender [52, 56, 58, 61, 69]; 16% considered the intersection between ethnicity and gender [52, 53, 56, 57]; 8% considered the intersection between education and gender [59, 69]; 8% considered the intersection between parental status and gender [52, 68]; 4% considered the intersection between pregnancy status and gender [57] and 4% considered the intersection between disability and gender [69]. Women with a higher level of education or income, women without children, able-bodied women, and white women were more typically associated with positive health outcomes. Results by age group were mixed. Overall, this suggests that the introduction of SUDMs risks exacerbating health inequalities and highlights the importance of disaggregating data by multiple demographic characteristics. These results are displayed in Fig. 3.

## Discussion

### Key Findings

This systematic review sought to understand what is currently known about the impact of SUDMs on women’s health and wellbeing, and what mechanistic pathways are outlined in existing literature from SUDMs to gendered health outcomes. We identified 25 papers which met our inclusion criteria, finding that the majority of the literature focuses on physical activity, and particularly, walking behavior. This aligns with policies that suggest reducing car-centrism in urban areas will lead to increased physical activity, with subsequent positive physical and mental health benefits [5, 70, 71]. However, evidence of pathways linking changes in physical activity to long-term mental and physical health outcomes in the context of SUDMs is comparatively lacking.

Recent policies promoting the introduction of SUDMs suggest that they may help to reduce health inequalities. However, within our search, any literature which considered health and wellbeing inequalities found that positive changes in physical, mental, and health behavioural outcomes were often not significant for multiply-disadvantaged groups, particularly women of colour. This emphasizes the need for an intersectional systems thinking approach to designing SUDMs and analysing their subsequent impact, as is encouraged within feminist urbanist and public health literature [13, 22, 32, 72].

Pathways outlined from SUDMs to health behavioural outcomes were principally related to safety and social reproductive labor, although opportunities for social interaction within conveniently located public spaces were also thought to play a role. Throughout the papers included in the review, there is an interesting conceptual distinction between safety as an outcome and safety as a mechanism to promote increased physical activity. Papers that observed increased physical activity for women within SUDMs often hypothesized that this was due to an increased sense of safety associated with changes to the built environment; whereas those papers which actually explored feelings of safety in relation to SUDMs found no significant changes [48, 65, 66]. There therefore seems to be a mechanistic gap in evidencing the link between SUDMs, perceived safety and walking behavior. Since safety is a key aspect of feminist urbanism, this gap could be explored in future research [27, 28].

Feminist urbanist literature suggests that women’s needs should be substantively represented in urban planning, thereby reflecting and easing the execution of the disproportionate amount of unpaid care work they undertake [25, 26]. To some extent, SUDMs seem to promote such substantive representation. This review found that proximity-based SUDMs in which women live closer to care infrastructure are associated with increased physical activity. This suggests that women take advantage of the improved access to care infrastructure by walking to destinations. However, cognisant of Marquet and Maciejewska’s warning, we do not intend for this result to reinforce essentialist associations between women and reproductive labor [22]. Further research could explore gender differences between men and women to understand whether SUDMs promote a more equitable balance of social reproductive labor between men and women.

Most commonly, papers did not explicitly address ‘feminist urbanism’ as a concept, but they did often focus on women’s daily travel patterns within urban environments, highlighting how these patterns vary according to different needs. For instance, some papers critiqued the tendency of compact development to reflect men’s travel patterns to the exclusion of women’s, speaking to the lack of women’s substantive representation within urban planning [69]. Meanwhile, others considered how our approaches to analysing walkability are inherently gendered, discovering in their research that women’s concepts of walkability vary significantly from the often-cited and widely utilized Walk Score [48].

### Research Gaps

The review has identified several research gaps in the literature in relation to SUDMs and women’s health and wellbeing. Firstly, the literature is dominated by health behaviors, with a minority of included studies exploring the relationship between urban models and women’s mental health and wellbeing, or physical health (44%). In particular, no studies considered pathways from reduced car use within SUDMs, lower particulate air pollution, and long-term rates of non-communicable diseases. Secondly, none of the included papers took an exclusively qualitative approach. There is therefore room for more qualitative exploration of women’s experiences of SUDMs and how this relates to their health and wellbeing. In particular, several papers suggest that increased physical activity in women is because they are able to conduct their ‘second shift’ of social reproductive labor with greater ease within SUDMs. However, none of the included papers explored what it is like for women to conduct this labor within SUDMs or related this to their mental health. Geographically, the literature predominantly originates in the US. The UK is notably missing from the included papers, despite commitments to X-MN policies and feminist city planning in recent years [18]. Finally, although Golan et al.’s paper found that safety was a baseline for walkability for women, none of the papers attempted to test the wider applicability of this knowledge and its relationship to women’s walking behavior [48].

### Limitations

There are some important limitations to consider when interpreting our findings. Although our only geographical criteria was that the research focused on urban spaces, most included papers originated in the US. This arguably limits the transferability of our findings to other countries, particularly those which are less car-centric than the US. However, there were no notable differences in outcomes across similar study designs in different countries, suggesting that the pathways outlined for US-centric studies were consistent with those outside of the US. Relatedly, since we only included papers published in English, it is likely that papers exploring SUDMs in non-English-speaking countries have been missed. Finally, the protocol for this review was not registered.

## Conclusion

SUDMs are typically associated with positive physical health and health behavioural outcomes for women. These included increased physical activity, healthier weight, reduced smoking behavior, and improved pregnancy outcomes. Evidence related to mental health outcomes is limited and mixed. Key mechanisms related to women’s increased physical activity within SUDMs are the ability to conduct their ‘second shift’ of social reproductive labor with greater ease, feeling safer within more walkable environments, and having increased opportunities for social interaction within their local residential area. However, existing social inequalities appear to persist within SUDMs, with results varying according to age, income and ethnicity. Overall, this suggests that SUDMs do offer an opportunity for cities to better reflect the distinct needs of women, leading to positive health outcomes, but reinforces the need for intersectional research to ensure the needs of all women are recognized.

## Data Availability

The data extracted and analysed were deposited to the OSF online data repository. They are available at the following URL: https://osf.io/vdja6/?view_only=5f9643a8b3a845739905cac38c9a9aef

## Appendix 1. PRISMA 2020 main checklist [35]

**Table:**

Topic	No	Item	Location where item is reported
**TITLE**			
**Title**	1	Identify the report as a systematic review	1
**ABSTRACT**			
**Abstract**	2	See the PRISMA 2020 for Abstracts checklist	
**INTRODUCTION**			
**Rationale**	3	Describe the rationale for the review in the context of existing knowledge	3–7
**Objectives**	4	Provide an explicit statement of the objective(s) or question(s) the review addresses	7
**METHODS**			
**Eligibility criteria**	5	Specify the inclusion and exclusion criteria for the review and how studies were grouped for the syntheses	8–10
**Information sources**	6	Specify all databases, registers, websites, organisations, reference lists and other sources searched or consulted to identify studies. Specify the date when each source was last searched or consulted	9
**Search strategy**	7	Present the full search strategies for all databases, registers and websites, including any filters and limits used	33–36
**Selection process**	8	Specify the methods used to decide whether a study met the inclusion criteria of the review, including how many reviewers screened each record and each report retrieved, whether they worked independently, and if applicable, details of automation tools used in the process	11
**Data collection process**	9	Specify the methods used to collect data from reports, including how many reviewers collected data from each report, whether they worked independently, any processes for obtaining or confirming data from study investigators, and if applicable, details of automation tools used in the process	11
**Data items**	10a	List and define all outcomes for which data were sought. Specify whether all results that were compatible with each outcome domain in each study were sought (e.g. for all measures, time points, analyses), and if not, the methods used to decide which results to collect	11
	10b	List and define all other variables for which data were sought (e.g. participant and intervention characteristics, funding sources). Describe any assumptions made about any missing or unclear information	11
**Study risk of bias assessment**	11	Specify the methods used to assess risk of bias in the included studies, including details of the tool(s) used, how many reviewers assessed each study and whether they worked independently, and if applicable, details of automation tools used in the process	11
**Effect measures**	12	Specify for each outcome the effect measure(s) (e.g. risk ratio, mean difference) used in the synthesis or presentation of results	12
**Synthesis methods**	13a	Describe the processes used to decide which studies were eligible for each synthesis (e.g. tabulating the study intervention characteristics and comparing against the planned groups for each synthesis (item 5))	12
	13b	Describe any methods required to prepare the data for presentation or synthesis, such as handling of missing summary statistics, or data conversions	NA
	13c	Describe any methods used to tabulate or visually display results of individual studies and syntheses	12
	13d	Describe any methods used to synthesize results and provide a rationale for the choice(s). If meta-analysis was performed, describe the model(s), method(s) to identify the presence and extent of statistical heterogeneity, and software package(s) used	12
	13e	Describe any methods used to explore possible causes of heterogeneity among study results (e.g. subgroup analysis, meta-regression)	NA
	13f	Describe any sensitivity analyses conducted to assess robustness of the synthesized results	NA
**Reporting bias assessment**	14	Describe any methods used to assess risk of bias due to missing results in a synthesis (arising from reporting biases)	NA
**Certainty assessment**	15	Describe any methods used to assess certainty (or confidence) in the body of evidence for an outcome	11
**RESULTS**			
**Study selection**	16a	Describe the results of the search and selection process, from the number of records identified in the search to the number of studies included in the review, ideally using a flow diagram	13
	16b	Cite studies that might appear to meet the inclusion criteria, but which were excluded, and explain why they were excluded	NA
**Study characteristics**	17	Cite each included study and present its characteristics	7
**Risk of bias in studies**	18	Present assessments of risk of bias for each included study	7
**Results of individual studies**	19	For all outcomes, present, for each study: (a) summary statistics for each group (where appropriate) and (b) an effect estimate and its precision (e.g. confidence/credible interval), ideally using structured tables or plots	7
**Results of syntheses**	20a	For each synthesis, briefly summarize the characteristics and risk of bias among contributing studies	7
	20b	Present results of all statistical syntheses conducted. If meta-analysis was done, present for each the summary estimate and its precision (e.g. confidence/credible interval) and measures of statistical heterogeneity. If comparing groups, describe the direction of the effect	7
	20c	Present results of all investigations of possible causes of heterogeneity among study results	18–22
	20d	Present results of all sensitivity analyses conducted to assess the robustness of the synthesized results	NA
**Reporting biases**	21	Present assessments of risk of bias due to missing results (arising from reporting biases) for each synthesis assessed	NA
**Certainty of evidence**	22	Present assessments of certainty (or confidence) in the body of evidence for each outcome assessed	NA
**DISCUSSION**			
**Discussion**	23a	Provide a general interpretation of the results in the context of other evidence	24–26
	23b	Discuss any limitations of the evidence included in the review	26
	23c	Discuss any limitations of the review processes used	27
	23d	Discuss implications of the results for practice, policy, and future research	27
**OTHER INFORMATION**			
**Registration and protocol**	24a	Provide registration information for the review, including register name and registration number, or state that the review was not registered	27
	24b	Indicate where the review protocol can be accessed, or state that a protocol was not prepared	27
	24c	Describe and explain any amendments to information provided at registration or in the protocol	NA
**Support**	25	Describe sources of financial or non-financial support for the review, and the role of the funders or sponsors in the review	28
**Competing interests**	26	Declare any competing interests of review authors	28
**Availability of data, code and other materials**	27	Report which of the following are publicly available and where they can be found: template data collection forms; data extracted from included studies; data used for all analyses; analytic code; any other materials used in the review	28

## Appendix 2. Search strategy

Search date: 24/02/2025

**ASSIA**

**Table:**

1	noft((20-min neighborhood) OR (20-min neighborhood) OR (20-min city) OR (15-min neighborhood) OR (15-min neighborhood) OR (15-min city) OR (10-min neighborhood) OR (10-min neighborhood) OR (10-min city) OR (1-min city) OR (1-min neighborhood) OR (1-min neighborhood) OR (x-minute city) OR (x-minute neighborhood) OR (x-minute neighborhood) OR (compact city) OR (garden city) OR (smart city) OR (transit oriented development) OR (transit-oriented development) OR (new urbanism) OR (eco-urbanism) OR (eco urbanism) OR (walkable city) OR (walkable neighborhood) OR (walkable neighborhood) OR (active city) OR (liveable neighborhood) OR (liveable neighborhood) OR (liveable city) OR (superblock) OR (super-block) OR (vital neighborhood) OR (vital neighborhood) OR (densification) OR (isobenefit urban) OR (iso-benefit urban) OR (city blocks) OR (lineal city) OR (Broadacre city) OR (polycentric city) OR (time geography) OR (traditional neighborhood design) OR (traditional neighborhood design) OR (human-scale city) OR (human scale city) OR (chronourbanism) OR (chrono-urbanism) OR (neighborhood unit plan) OR (neighborhood unit plan) OR (post-modern urbanism) OR (post modern urbanism)) AND PEER(yes) AND PUBYEAR < 2024	**2825**
2	Noft((female) OR (woman) OR (women) OR (girl) OR (sex) OR (gender)) AND PEER(yes)	**277,241**
3	noft((Health) OR (wellbeing) OR (mental health) OR (social interaction) OR (social isolation) OR (lonely) OR (loneliness) OR (mortality) OR (morbidity) OR (life expectancy) OR (DALYs) OR (QALYs) OR (physical activity) OR (healthy eating) OR (diet) OR (exercise) OR (safety) OR (morbidity) OR (obesity) OR (cardiovascular) OR (stroke) OR (bmi) OR (social cohesion) OR (depression) OR (mental disorder) OR (anxiety) OR (stress) OR (sense of belonging) OR (sense of community) OR (walk) OR (walking) OR (respiratory) OR (step count) OR (wheeling) OR (cycle) OR (cycling) OR (smoking)) AND PEER(yes)	**676,317**
4	((HIV) OR (chemotherapy))	
5	#1 and #2 and #3	**750**
6	#1 and #2 and #3 NOT #4	**536**

**Scopus**

**Table:**

**1**	(TITLE-ABS-KEY (“20*minute neighborhood”) OR TITLE-ABS-KEY (“20*minute neighborhood”) OR TITLE-ABS-KEY (“20*minute city”) OR TITLE-ABS-KEY (“15*minute neighborhood”) OR TITLE-ABS-KEY (“15*minute neighborhood”) OR TITLE-ABS-KEY (“15*minute city”) OR TITLE-ABS-KEY (“10*minute neighborhood”) OR TITLE-ABS-KEY (“10*minute neighborhood”) OR TITLE-ABS-KEY (“10*minute city”) OR TITLE-ABS-KEY (“1*minute neighborhood”) OR TITLE-ABS-KEY (“1*minute neighborhood”) OR TITLE-ABS-KEY (“1*minute city”) OR TITLE-ABS-KEY(“x*minute city”) OR TITLE-ABS-KEY(“x*minute neighborhood”) OR TITLE-ABS-KEY(“x*minute neighborhood”) OR TITLE-ABS-KEY (“compact city”) OR TITLE-ABS-KEY (“garden city”) OR TITLE-ABS-KEY (“smart city”) OR TITLE-ABS-KEY (“eco*urbanism”) OR TITLE-ABS-KEY (“transit*oriented development”) OR TITLE-ABS-KEY (“new urbanism”) OR TITLE-ABS-KEY (“walkable city”) OR TITLE-ABS-KEY (“walkable neighborhood”) OR TITLE-ABS-KEY (“walkable neighborhood”) OR TITLE-ABS-KEY (“transit*oriented development”) OR TITLE-ABS-KEY (“new urbanism”) OR TITLE-ABS-KEY (“active city”) OR TITLE-ABS-KEY (“liveable neighborhood”) OR TITLE-ABS-KEY (“liveable neighborhood”) OR TITLE-ABS-KEY (“liveable city”) OR TITLE-ABS-KEY (“vital neighborhood”) OR TITLE-ABS-KEY (“vital neighborhood”) OR TITLE-ABS-KEY (“densification”) OR TITLE-ABS-KEY (“neighborhood unit plan”) OR TITLE-ABS-KEY (“neighborhood unit plan”) OR TITLE-ABS-KEY (“post*modern urbanism”))	**97,645**
2	(TITLE-ABS-KEY (“female”) OR TITLE-ABS-KEY (“woman”) OR TITLE-ABS-KEY (“women”) OR TITLE-ABS-KEY (“girl”) OR TITLE-ABS-KEY (“sex”) OR TITLE-ABS-KEY (“gender”))	**13,184,307**
3	(TITLE-ABS-KEY (“health”) OR TITLE-ABS-KEY (“wellbeing”) OR TITLE-ABS-KEY (“mental health”) OR TITLE-ABS-KEY (“social isolation”) OR TITLE-ABS-KEY (“loneliness”) OR TITLE-ABS-KEY (“lonely”) OR TITLE-ABS-KEY (“mortality”) OR TITLE-ABS-KEY (“morbidity”) OR TITLE-ABS-KEY (“healthy eating”) OR TITLE-ABS-KEY (“diet”) OR TITLE-ABS-KEY (“physical activity”) OR TITLE-ABS-KEY (“exercise”) OR TITLE-ABS-KEY (“cycling”) OR TITLE-ABS-KEY (“walking”) OR TITLE-ABS-KEY (“life expectancy”) OR TITLE-ABS-KEY (“DALYs”) OR TITLE-ABS-KEY (“QALYs”) OR TITLE-ABS-KEY (“social interaction”) OR TITLE-ABS-KEY (“morbidity”) OR TITLE-ABS-KEY (“obesity”) OR TITLE-ABS-KEY (“stroke”) OR TITLE-ABS-KEY(“cardiovascular”) OR TITLE-ABS-KEY (“safety”) OR TITLE-ABS-KEY (“bmi”) OR TITLE-ABS-KEY (“social cohesion”) OR TITLE-ABS-KEY (“depression”) OR TITLE-ABS-KEY (“mental disorder”) OR TITLE-ABS-KEY (“anxiety”) OR TITLE-ABS-KEY (“stress”) OR TITLE-ABS-KEY (“sense of belonging”) OR TITLE-ABS-KEY(“sense of community”) OR TITLE-ABS-KEY (“walk”) OR TITLE-ABS-KEY (“respiratory”) OR TITLE-ABS-KEY (“walking”) OR TITLE-ABS-KEY (“step count”) OR TITLE-ABS-KEY (“cycling”) OR TITLE-ABS-KEY (“cycle”) OR TITLE-ABS-KEY (“wheeling”) OR TITLE-ABS-KEY (“smoking”))	**19,128,955**
4	**NOT (**TITLE-ABS-KEY (“ HIV”) OR TITLE-ABS-KEY (“chemotherapy”))	
5	**#1 and #2 and #3 (limited to English)**	**719**
6	**#1 and #2 and #3 and #4**	**445**

**Web of Science (SCIE & SSCI)**

**Table:**

**1**	TS = (“20-min neighborhood” OR “20-min neighborhood” OR “20-min city” OR “15-min neighborhood” OR “15-min neighborhood” OR “15-min city” OR “10-min neighborhood” OR “10-min neighborhood” OR “10-min city” OR “1-min neighborhood” OR “1-min neighborhood” OR “1-min city” OR “x-minute neighborhood” OR “x-minute neighborhood” OR “x-minute city” OR “20-min neighborhood” OR “20-min neighborhood” OR “20-min city” OR “15-min neighborhood” OR “15-min neighborhood” OR “15-min city” OR “1-min neighborhood” OR “1-min neighborhood” OR “1-min city” OR “x-min neighborhood” OR “x-min neighborhood” OR “x-min city” OR “compact city” OR “garden city” OR “smart city” OR “eco-urbanism” OR “eco urbanism” OR “transit-oriented development” OR “new urbanism” OR “walkable city” OR “walkable neighborhood” OR “walkable neighborhood” OR “active city” OR “liveable neighborhood” OR “liveable neighborhood” OR “liveable city” OR “vital neighborhood” OR “vital neighborhood” OR “densification” OR “neighborhood unit plan” OR “neighborhood unit plan” OR “post-modern urbanism”)	**43,419**
2	TS = (female OR woman OR women OR girl OR sex OR gender)	**3,508,830**
3	TS = (Health OR wellbeing OR “mental health” OR “social isolation” OR mortality OR morbidity OR “life expectancy” OR DALYs OR QALYs OR “physical activity” OR “healthy eating” OR diet OR exercise OR “social interaction” OR “social isolation” OR loneliness OR safety OR obesity OR stroke OR cardiovascular OR bmi OR “social cohesion” OR depression OR respiratory OR “mental disorder” OR anxiety OR stress OR “sense of belonging” OR “sense of community” OR walk OR walking OR “step count” OR cycle OR cycling OR wheeling OR smoking)	**10,566,116**
4	**#1 and #2 and #3 and English**	**91**

**Medline (OVID)**

**Table:**

1	(“20-min neighborhood$” or “20-min neighborhood$” or “20-min cit$” or “15-min neighborhood$” or “15-min neighborhood$” or “15-min cit$” or “1-min neighborhood$” or “1-min neighborhood$” or “1-min cit$” or “x-minute neighborhood$” or “x-minute neighborhood$” or “x-minute cit$” or “20-min neighborhood$” or “20-min neighborhood$” or “20-min cit$” or “15-min neighborhood$” or “15-min neighborhood$” or “15-min cit$” or “1-min neighborhood$” or “1-min neighborhood$” or “1-min cit$” or “x-min neighborhood$” or “x-min neighborhood$” or “x-min cit$” or “compact cit$” or “garden cit$” or “smart cit$” or “eco-urbanism” or “transit-oriented development$” or “new urbanism” or “walkable cit$” or “walkable neighborhood$” or “walkable neighborhood$” or “active cit$” or “liveable neighborhood$” or “liveable neighborhood$” or “liveable cit$” or “vital neighborhood$” or “vital neighborhood$” or “urban densification” or “neighborhood unit plan$” or “neighborhood unit plan$” or “post-modern urbanism”).ab,ti **Limited to pre-2023 publication and English language**	**1840**
2	(female or woman or women or girl or sex or gender).ab,ti **Limited to pre-2023 publication and English language**	**3047910**
3	(Health or wellbeing or “mental health” or “social isolation” or mortality or morbidity or “life expectancy” or DALYs or QALYs or “physical activity” or “healthy eating” or diet or exercise or “social interaction” or “social isolation” or loneliness or safety or morbidity or obesity or stroke or cardiovascular or bmi or “social cohesion” or depression or “mental disorder” or respiratory or anxiety or stress or “sense of belonging” or “sense of community” or walk or walking or “step count” or cycling or cycle or wheeling or smoking).ab,ti **Limited to pre-2023 publication and English language**	**7016604**
**4**	**#1 and #2 and #3**	**99**

## Appendix 3. Definition of SUDM terms

**Table:**

Model	Definition	Relation to sustainability
*Compact City* [73]	prioritizes development within higher density, multi-use spaces, linked by city-wide public transport networks facilitating intra-neighborhood connectivity	Compact multi-use spaces reduce the need for travel, and the presence of efficient public transport networks reduce the need for private vehicle use
*Densification* [74]	prioritizes increasing population densities and employment, often achieved by'building upwards'and reusing brownfield land	Seeks to minimize urban sprawl and in doing so preserve existing greenspace and biodiversity, whilst reducing the need to commute using private vehicles
*New Urbanism* [75]	prioritizes neighborhood-level development, ensuring residents have access to a diverse range of workplaces, retail facilities, schools, greenspace and public services which they might need to access on a daily basis within walking distance of their home	By developing proximate access to amenities within neighborhoods, residents are less likely to rely upon their cars
*Smart City* [76]	City development model which prioritizes the integration of digital sensing technologies within urban infrastructure linked to central analysis and control systems. Seen as offering potential for more efficient resource use and greater public safety	By making public transport more efficient and active travel safer and more convenient, smart city developments have the potential to incentivize reduced use of private vehicles
*Transit-Oriented Development* [77]	prioritizes improving the proximity of jobs, housing, services and amenities to public transport networks	Encourages the use of public transport, rendering private ownership of vehicles less necessary
*Walkable neighborhood/City* [78]	prioritizes improving the quality and connectivity of the pedestrian environment and improving proximity to amenities	By improving the environment in which people walk and by developing more convenient routes, urban residents are incentivized to walk or wheel more, and reduce their use of private vehicles
*X-Minute neighborhood/City* [16]	prioritizes improving access to commonly-utilized amenities within walking distance from residents'homes; often accompanied by the development of active travel infrastructure to facilitate non-motorized vehicular access	By developing proximate access to amenities within neighborhoods and by improving the environment in which people engage in active travel, residents are less likely to rely upon their cars
